# Ischemic Preconditioning Modulates the Peripheral Innate Immune System to Promote Anti-Inflammatory and Protective Responses in Mice Subjected to Focal Cerebral Ischemia

**DOI:** 10.3389/fimmu.2022.825834

**Published:** 2022-03-11

**Authors:** Diana Amantea, Daniele La Russa, Marialaura Frisina, Francesca Giordano, Chiara Di Santo, Maria Luisa Panno, Giuseppe Pignataro, Giacinto Bagetta

**Affiliations:** ^1^ Department of Pharmacy, Health and Nutritional Sciences, University of Calabria, Rende (CS), Italy; ^2^ Department of Neuroscience, Reproductive and Odontostomatological Sciences, “Federico II” University, Naples, Italy

**Keywords:** immune system, ischemic stroke, ischemic tolerance, preconditioning, miRNA

## Abstract

The development of tolerance triggered by a sublethal ischemic episode (preconditioning, PC) involves a complex crosstalk between neurons, astrocytes and microglia, although the role of the peripheral immune system in this context is largely unexplored. Here, we report that severe cerebral ischemia caused by transient middle cerebral artery occlusion (MCAo) in adult male mice elevates blood counts of inflammatory neutrophils and monocytes, and plasma levels of miRNA-329-5p. These inflammatory responses are prevented by ischemic PC induced by 15 min MCAo, 72h before the severe insult (1h MCAo). As compared with sham-operated animals, mice subjected to either ischemic PC, MCAo or a combination of both (PC+MCAo) display spleen contraction. However, protein levels of Ym1 (a marker of polarization of myeloid cells towards M2/N2 protective phenotypes) are elevated only in spleen from the experimental groups PC and PC+MCAo, but not MCAo. Conversely, Ym1 protein levels only increase in circulating leukocytes from mice subjected to 1h MCAo, but not in preconditioned animals, which is coincident with a dramatic elevation of Ym1 expression in the ipsilateral cortex. By immunofluorescence analysis, we observe that expression of Ym1 occurs in amoeboid-shaped myeloid cells, mainly representing inflammatory monocytes/macrophages and neutrophils. As a result of its immune-regulatory functions, ischemic PC prevents elevation of mRNA levels of the pro-inflammatory cytokine interleukin (IL)-1β in the ipsilateral cortex, while not affecting IL-10 mRNA increase induced by MCAo. Overall, the elevated anti-inflammatory/pro-inflammatory ratio observed in the brain of mice pre-exposed to PC is associated with reduced brain infarct volume and ischemic edema, and with amelioration of functional outcome. These findings reaffirm the crucial and dualistic role of the innate immune system in ischemic stroke pathobiology, extending these concepts to the context of ischemic tolerance and underscoring their relevance for the identification of novel therapeutic targets for effective stroke treatment.

## Introduction

Ischemic stroke is a major cause of death and serious long-term disability worldwide (1). Reperfusion by pharmacological thrombolysis or mechanical thrombectomy represents the only acute treatment approved (2–4), highlighting the urgent need for effective neuroprotective strategies. Interestingly, ischemic stroke patients who previously suffered a recent transient ischemic attack show better outcomes compared to those who did not, suggesting that ischemic tolerance may develop following a brief cerebral insult (5–10). In fact, ischemic tolerance consists in endogenous neuroprotective mechanisms that can be triggered by a non-invasive, sublethal ischemic episode (namely, preconditioning, PC) that produces resilience to subsequent severe ischemia (11, 12). The latter involves gene activation and *de novo* protein synthesis that reprogram the brain transcriptional response to ischemia and prompt a complex and still unresolved protective phenotype (13–15). Mechanisms of ischemic tolerance include preservation of mitochondrial function and ionic homeostasis, attenuation of excitotoxic and inflammatory processes, suppression of cell death pathways, augmentation of neurotrophic factors and vascular remodelling (16–20). Ischemic PC was initially thought to be mediated by adaptive responses triggered almost exclusively in neurons (21–23). However, more recent evidence has highlighted that innate immune pathways participate in the development of tolerance through Toll-like receptors (TLRs), Type I interferon (INF) signalling and genomic reprogramming of microglia towards reparative and protective phenotypes (24–27). In fact, in addition to their detrimental roles, microglia could also exert beneficial functions in stroke and/or contribute to protection by ischemic PC, likely through activation of CXCR1 receptor by neuronally-derived fractalkine (25, 28–31). Thus, current knowledge highlights that the neuro-immune response to ischemic PC involves a complex crosstalk between microglia, astrocytes and neurons (32–34); however, the role of the peripheral immune system in the establishment of ischemic tolerance is largely unexplored (35–37). The relevance of the latter concept stems from the crucial role played by peripheral immune mediators in the evolution of ischemic brain damage (38, 39) and from the numerous data demonstrating immune-regulatory mechanisms shared by diverse (i.e., ischemic, inflammatory/endotoxin, pharmacological, etc.) conditioning stimuli that might likely affect responses occurring outside the brain (27, 40–43). For example, adoptive transfer of monocytes isolated from mice preconditioned with low-dose lipopolysaccharide (LPS) into naive mice reduced MCAo-induced brain injury. These protective monocytes are generated in the spleen and traffic to the brain and meninges, where they suppress post-ischemic inflammation and neutrophil influx (41). Thus, the identification of novel pharmacological targets in the periphery is rather intriguing, as it offers the possibility to target peripheral responses to tune the progression of cerebral damage. Therefore, the major aim of the present work was to characterize the peripheral innate immune response triggered by ischemic PC in mice subjected to transient middle cerebral artery occlusion (MCAo).

## Experimental Procedures

### Animals

Experimental procedures were performed on 8-10 weeks old, C57Bl/6J male mice (Charles River, Calco, Como, Italy), housed under controlled environmental conditions (i.e., temperature of 22°C, relative humidity of 65% and 12 h light:12 h dark cycle), with free access to food and water.

The *in vivo* procedures were performed in mice housed for at least two weeks upon arrival, following the guidelines of the Italian Ministry of Health (DL 26/2014), in accordance with the 2010/63/UE European Directive, and the protocols were approved (n. 975/2017-PR and 701/2020-PR) by the Committee set by the Ministry of Health at the National Institute of Health (Rome). The study was realized observing the ARRIVE guidelines (44) and maximal effort was made to reduce the number of animals used and their suffering.

Mice were randomly allocated to the following experimental groups:

1) SHAM: sham surgery 24h before sacrifice;
2) PC: 15-min MCAo followed by 72h of reperfusion;
3) MCAo: 1-h MCAo (preceded by SHAM surgery 72h before) followed by 24h of reperfusion;
5) PC + MCAo: 15-min MCAo followed, 72h later, by 1-h MCAo and 24h of reperfusion.

The minimum sample size was evaluated using an *a priori* power analysis adjusted to obtain a power of 80% at a significance level of 0.05 (OpenEpi software 3.01, Open Source Statistics for Public Health). On the basis of our earlier experience with the MCAo model, we hypothesized a difference in ischemic volume between mice subjected to MCAo and mice exposed to a neuroprotective procedure (i.e., ischemic PC) of about 30 mm^3^ (approximately 30% reduction of infarct size) and a variability (standard deviation) of 15. This led to an estimated minimum sample size of 4 animals per experimental group.

### Surgical Procedure for MCAo

Focal cerebral ischemia was produced by proximal occlusion of the middle cerebral artery, using a previously described technique (45, 46). Briefly, the external carotid artery was isolated in mice anaesthetized with isoflurane (1.5-2%, vaporized in air), and a silicone-coated nylon filament (diameter: 0.23 mm, Doccol Corporation, Redlands, CA, USA) was introduced into the internal carotid artery up to the Willis circle of Willis to occlude the origin of the middle cerebral artery proven by a >70% cortical cerebral blood flow (CBF) reduction (47). Six animals were excluded from the study because of unsuccessful MCAo, i.e., less than 70% reduction of CBF.

Ischemic PC was reproduced using a well-established paradigm (12, 17, 25, 48, 49), consisting in a brief (15-min) MCAo, followed by a 72-h period of recovery and reperfusion, to be applied before the more severe ischemia (i.e., 1h MCAo followed by 24h of reperfusion, PC + MCAo). SHAM-control conditions were reproduced by performing identical surgical procedures except for filament insertion into the middle cerebral artery. Eight animals died during or early after surgery and were excluded from the study.

### Assessment of Infarct Size, Edema and Neurological Deficits

Cerebral ischemic damage was assessed after 24 h of reperfusion (following 1h MCAo, preceded or not by PC). To this end, frozen brains were sectioned by a cryostat into fifteen, 20 μm-thick coronal slices, at 0.5 mm intervals from the frontal pole. The slices were mounted on glass slides and stained with cresyl violet. Infarct (pale) areas were assessed by an investigator blinded to treatment allocation using an image analysis software (ImageJ, version 1.30), and to calculate infarct size and edema volume (mm^3^) as previously described (50).

Neurological deficits were assessed 24h after MCAo or SHAM surgery, or 72h after PC by using the dichotomized De Simoni composite neuroscore that allows to evaluate the general and focal neurological dysfunctions caused by the ischemic insult (51, 52). Briefly, total score ranges from 0 (healthy) to 56 (the worst performance in all the 13 categories) and represents the sum of 6 general deficits (fur [0-2], ears [0-2], eyes [0-4], posture [0-4], spontaneous activity [0-4], and epileptic behavior [0-12]) and 7 focal deficits (body asymmetry [0-4], gait [0-4], climbing [0-4], circling behavior [0-4], forelimb symmetry [0-4], compulsory circling [0-4], and whisker response [0-4].

### Western Blot Analysis

Ipsilateral (ischemic) and contralateral frontoparietal cortices (3.2 to -3.8 mm from Bregma) (53) and spleens were rapidly dissected 24h after MCAo or SHAM surgery, or 72h after PC, and homogenized in ice-cold RIPA buffer containing protease inhibitor cocktail (Sigma-Aldrich, Milan, Italy) and centrifuged for 20 min at 20817 g at 4°C. Protein concentration was quantified in supernatants (Bradford method, PanReac AppliChem, ITW Reagents) and equal amounts (40 μg) were heated for 5 min in Laemmli buffer (Sigma-Aldrich, Milan, Italy), separated by sodium dodecyl sulphate polyacrylamide gel electrophoresis (SDS-PAGE) in a Bio-Rad Mini Pro-tean IIIand, then electroblotted onto nitrocellulose membrane (NitroBind, Maine Manufacturing, Maine, U.S.A.) using a mini trans-blot (Bio-Rad Laboratories, Hercules, CA, U.S.A.). After blocking with 5% non-fat milk in 0.05% Tween-20 TRIS-buffered saline (TBS-T), the blots were incubated overnight, at 4°C, with the following primary antibodies: rabbit anti-Ym1 (1:1000; 60130, StemCell Technologies) and mouse anti-β-actin (1:1.000; sc-69879, Santa Cruz Biotechnology, Inc.), followed by species-specific peroxidase-linked secondary antibodies (1:2.000; Santa Cruz Biotechnology, Inc.) for 1 h at room temperature. Immunodetection of protein bands was performed with enhanced chemiluminescence kit (Western Blotting Luminol Reagent, Santa Cruz Biotechnology, Inc) followed by exposure to X-ray films (Ultracruz Autoradiography Film, Santa Cruz Biotechnology, Inc). ImageJ software (National Institutes of Health, Bethesda, MD) was used for densitometric analysis of the bands.

### Immunofluorescence

Immunohistochemistry was performed on paraformaldehyde-fixed brains and spleens, dissected 24h after 1-h MCAo, and sectioned into 40 µm-thick slices. Coronal brain slices were collected at the level of the territory perfused by the middle cerebral artery (1.18 to -0.10 mm from Bregma) (53). Using a previously reported procedure (54, 55), the sections were incubated with the following primary antibodies: rabbit anti mouse Ym1 (1:100, StemCell Technologies, UK) to label alternatively-activated myeloid cells (56, 57); rat anti mouse Ly-6B.2 (1:200; clone 7/4; AbD Serotec, Kidlington, UK) to label Ly-6G^+^ neutrophils and Ly-6C^+^ inflammatory monocytes/macrophages (58–60); rat anti-mouse Ly-6G (1:200; clone 1A8; BD Pharmingen, Italy) to label neutrophils; rat anti-mouse Ly-6C (1:100; clone HK1.4; Biolegened, San Diego, USA); rat anti mouse CD11b (1:200; clone M1/70.15; AbD Serotec, Kidlington, UK) to label myeloid cells (comprising microglia, monocytes/macrophages, neutrophils and dendritic cells); goat anti-Iba1 (Thermo Fisher Scientific) to label microglia/macrophages (61). Primary antibodies were labelled with appropriate secondary antibodies conjugated with AlexaFluor 488 or AlexaFluor 568 (1:200 dilution; Invitrogen, Thermo Fisher Scientific, Italy); whereas, nuclei were counterstained with 4’,6-diamidino-2-phenylindole (DAPI, 1:500; Sigma-Aldrich, Milan, Italy). Fluorescence was examined under a confocal laser scanning microscope (Fluoview FV300, Olympus) equipped with a dedicated software module (cellSens) for image analysis.

To quantify CD11b immunopositive myeloid cells, three coronal brain sections were collected from each brain (n= 4 mice per experimental group) at 0.98, 0.38 and -0.22 mm from bregma, corresponding to the middle cerebral artery territory. Digitized images were acquired under identical microscope settings and cells were counted off-line, using ImageJ software, in 3-4 different optic fields of the confocal images (acquired through the 20× objective) of the ispilateral frontoparietal cortex. For each optic field, the total number of DAPI+ cells labelled for CD11b were counted and expressed as CD11b+ cells/0.31 mm^2^.

### Flow Cytometry

The expression of specific cell surface markers was assessed in leukocytes isolated from mouse blood samples collected in K_3_EDTA-containing tubes as previously reported (17). Briefly, after separating plasma by centrifugation at 300*g* for 10 min, the pellet was resuspended 2-3 times in BD Pharm Lyse™ (BD Bioscience) until complete erythrocyte lysis, and then incubated with anti-mouse CD16/32 antibody (1:50, TruStainFcX™, Biolegend, San Diego, CA, USA) to block non-specific binding to the Fc receptor. The samples were then incubated for 45 min at 4°C with the following PE/Dazzle594-labelled primary antibodies (Biolegend, San Diego, CA, USA): rat anti-mouse Ly-6G (1.5:100; #127648) or rat anti-mouse Ly-6C (1.5:100; #128044). After washing, the samples were analysed on a FACScan flow cytometer (Becton Dickinson, Mountain View, CA, USA) using CellQuest software. Neutrophils and monocytes populations were determined both by morphological features (i.e., forward and side scatter, FSC *vs.* SSC) and relative expression of Ly-6C and Ly-6G. Neutrophils were identified as FSC^high^/SSC^high^ events expressing Ly-6G; whereas, monocytes were identified as Ly-6C^+^/Ly-6G^-^ events after gating for FSC^high^/SSC^med^ population.

### Quantitative Polymerase Chain Reaction (PCR)

Quantitative real-time PCR analysis was performed on blood samples and on ipsilateral and contralateral brain cortices collected 24 h after 1h-MCAo (preceded or not by PC) or SHAM surgery, or 72 h after PC.

Total RNA was isolated from brain tissue using TRIzol Reagent (Thermo Fisher Scientific, MA, USA) according to the manufacturer’s protocol, and then dissolved in RNase-free water to determine concentration and quality using a Nanodrop 2000 spectrophotometer (Thermo Fisher). For each sample, 1 µg of total RNA was used for reverse transcription (RT) with the High Capacity RNA to cDNA kit (#4387406l, Applied Biosystems), following supplier’s instructions. PCR reactions were performed on the StepOnePlus Real-Time PCR system (Thermo Fisher) Primers for *interleukin(IL)-1β* (Mm00434228_m1), *IL-10* (Mm01288386_m1) and the housekeeping reference gene *glyceraldehyde 3-phosphate dehydrogenase* (*GAPDH*; Mm99999915_g1) were purchased from Thermo Fisher and quantification of gene expression was performed by the comparative cycle threshold (Ct) method.

Plasma was separated from blood samples, collected in EDTA Vacutainer^®^ tubes, by centrifugation at 1.500*g* for 15 min at 4°C, followed by centrifugation of the supernatant at 12.000*g* for 15 min at 4°C to pellet any debris and insoluble components. MicroRNA was isolated from of plasma samples using the miRNeasy Serum/Plasma Kit (Qiagen, Inc., Valencia, CA, USA) using *A. Thaliana* miR-159a (478411_mir, Thermo Fisher) as spike-in control. cDNA was synthetized by using TaqMan Advanced miRNA Assays Kit (Thermo Fisher) and miRNAs expression was detected by quantitative real-time PCR. Relative expression level of *miR-329-5p* (mmu482631_mir, Thermo Fisher) was calculated using the comparative Ct method and normalized to the expression of *miR-669c-3p* (mmu483332_mir, Thermo Fisher) that remained constant in all the samples analysed.

### Statistical Analysis

Data are expressed as scatter plots along with mean ± 95% confidence interval (CI) for quantitative variables, or medians with interquartile range for categorical ordinal variables (i.e., neuroscore). Data were subjected to statistical analysis using Graph-Pad Prism software for Windows (version 6.0, GraphPad Software, San Diego, CA) and Statistic Kingdom calculator (http://www.statskingdom.com, Melbourne, Australia) applying a fixed-effect statistical model. Comparisons between multiple experimental groups were performed using one- or two-way ANOVA followed by Tukey or Bonferroni post-tests, respectively. The neurological deficit scores, being ordinal in nature, were analyzed using the Kruskal-Wallis test followed by Bonferroni’s correction method for multiple comparisons. Values of P < 0.05 were considered to be significant.

## Results

**Figure 1:**
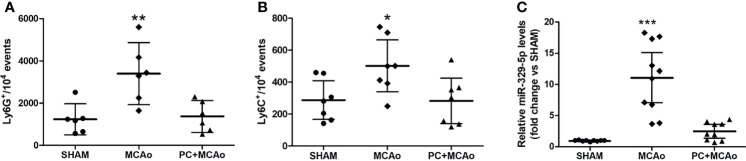
Ischemic PC attenuates the systemic inflammatory status caused by severe ischemia. **(A)** Circulating Ly-6G+ neutrophils, **(B)** Ly-6C+ monocytes and **(C)** plasma miR-329-5p levels in mice subjected to SHAM surgery, 1h MCAo preceded by sham surgery (MCAo) or by ischemic PC (15min MCAo) 72h before (PC+MCAo). *P = 0.0463 vs SHAM and P = 0.0412 vs PC+MCAo, **P = 0.0049 vs sham and P = 0.0080 vs PC+MCAo, ***P = 0.000003 vs SHAM and P = 0.00004 vs PC+MCAo (one-way ANOVA followed by Tukey’s *post-hoc* test, n=6-10 mice per experimental group).

In order to assess whether ischemic PC affects the peripheral inflammatory status, we measured the number of circulating Ly-6G^+^ neutrophils and Ly-6C^+^ monocytes in mice subjected to transient MCAo preceded or not by ischemic PC (i.e., 15-min MCAo) 72h before. As compared with SHAM surgery, 1h MCAo resulted in significant elevation of blood inflammatory neutrophils and monocytes measured 24h after reperfusion. Both effects were prevented in mice pre-exposed to ischemic PC (**Figure 1A**). Regarding monocyte subtypes, we observed that both Ly-6C^high^ and Ly-6C^low^ monocytes were reduced (by 35.71% and 47.06%, respectively) in the blood of preconditioned mice as compared with the MCAo group (data not shown). We next assessed modulation of plasma levels of miR-329-5p, identified by reverse target prediction analysis based on its potential to regulate inflammatory and immune mediators (e.g., TLR-4, IL-1 and TNF-related signaling pathways) involved in ischemia and PC. As shown in **Figure 1**, we originally observed that transient focal ischemia was associated with a significant elevation of miR-329-5p plasma levels that was abolished when mice were previously exposed to the PC stimulus. These findings suggest that a brief (i.e., 15-min), sublethal cerebral ischemia may exert beneficial effects by attenuating the peripheral inflammatory response evoked by a more severe ischemic insult.

**Figure 2:**
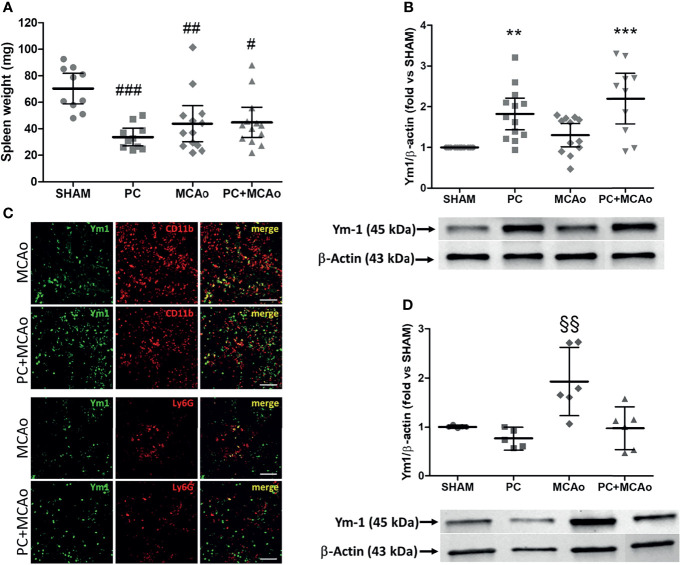
Ischemic PC does not affect spleen contraction induced by MCAo, while it elevates expression of the M2 marker Ym1 in spleen but not in circulating leukocytes. **(A)** Spleen weight and protein expression levels of Ym1 in spleen **(B)** and in circulating leukocytes **(D)** of mice subjected to SHAM surgery, ischemic PC (15 min MCAo followed by 72h of reperfusion), 1h MCAo followed by 24h of reperfusion (MCAo) or PC+MCAo. **(C)** Representative immunofluorescence images showing Ym1 expression (green fluorescence) in CD11b immunopositive myeloid cells and Ly-6G immunopositive neutrophils (red fluorescence) in the spleen parenchyma of mice undergone 1h MCAo preceded (72h before) by sham surgery (MCAo) or by ischemic PC (PC+MCAo). Scale bars = 50 μm. Data were analysed by one-way ANOVA followed by Tukey’s *post-hoc* test [**(A)** ^#^P = 0.0083, ^##^P = 0.0057 and ^###^P = 0.0002 vs SHAM, n = 10-13 mice per experimental group; **(B)** **P = 0.0046 vs SHAM, ***P = 0.0001 vs SHAM and P = 0.0037 vs MCAo, n = 8-13 mice per experimental group; **(D)** ^§§^P = 0.0098 vs SHAM, P = 0.0013 vs PC and P = 0.0051 vs PC+MCAo, n = 5-6 mice per experimental group].

Given the crucial role of the spleen in orchestrating the immune response occurring in the periphery and in the brain following ischemia (41, 62, 63), we investigated the effects of ischemic PC on this secondary lymphoid organ. Under our experimental conditions, spleen contraction occurred in mice subjected to either ischemic PC, MCAo or a combination of both (**Figure 2**). However, only the experimental groups PC and PC+MCAo (but not MCAo) showed increased protein expression of the M2 marker Ym1 in spleen homogenates as compared with tissue from sham-operated animals (**Figure 2**). Immunofluorescence analysis revealed that Ym1 was expressed in CD11b immunopositive myeloid cells, representing amoeboid-shaped monocytes and Ly-6G immunopositive neutrophils (**Figure 1**). Conversely, elevation of Ym1 expression was observed only in circulating leukocytes from mice subjected to 1h MCAo (**Figure 2**), despite its elevation in the spleen of mice belonging to the other experimental groups as compared to sham (**Figure 2**).

**Figure 3:**
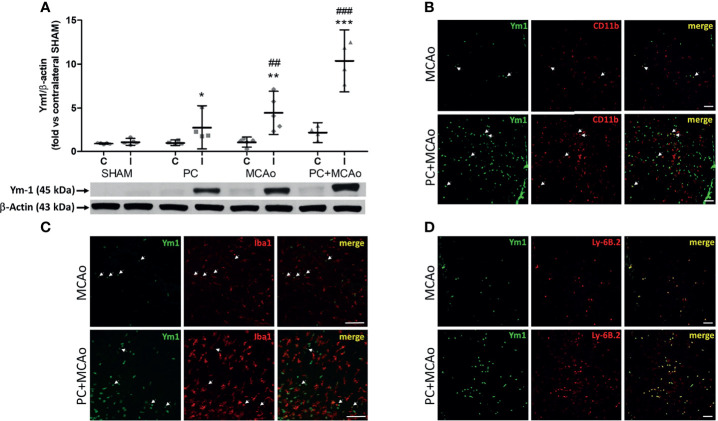
Ischemic PC potentiates the elevation of the M2/N2 marker Ym1 caused by 1h MCAo in myeloid cells of the ischemic cortex of mice. **(A)** Western blotting analysis of Ym1 expression in tissue homogenates from the contralateral (C) and ipsilateral (ischemic, I) cortex of mice subjected to SHAM surgery, ischemic PC (15 min MCAo followed by 72h of reperfusion), 1h MCAo followed by 24h of reperfusion (MCAo) or PC+MCAo. *P = 0.0479, **P = 0.0079 and ***P = 0.0004 vs corresponding contralateral; ^##^P = 0.0059 and ^###^P = 0.0003 vs ipsilateral SHAM (two-way ANOVA followed by Bonferroni post-test, n=4-5 mice per experimental group). Representative immunofluoresce images showing Ym1 expression (green fluorescence) in CD11b immunopositive myeloid cells [red fluorescence in **(B)**], Iba1 immunopositive microglia/macrophages [red fluorescence in **(C)**] or Ly-6B.2 immunopositive myeloid cells [granulocytes and monocytes/macrophages, red fluorescence in **(D)**] in the cortex of mice undergone 1h MCAo preceded (72h before) by sham surgery (MCAo) or by ischemic PC (PC+MCAo). Arrows indicate co-localization of Ym1 with CD11b or Iba1 in amoeboid-shaped cells. Scale bars = 100 μm.

**Figure 4:**
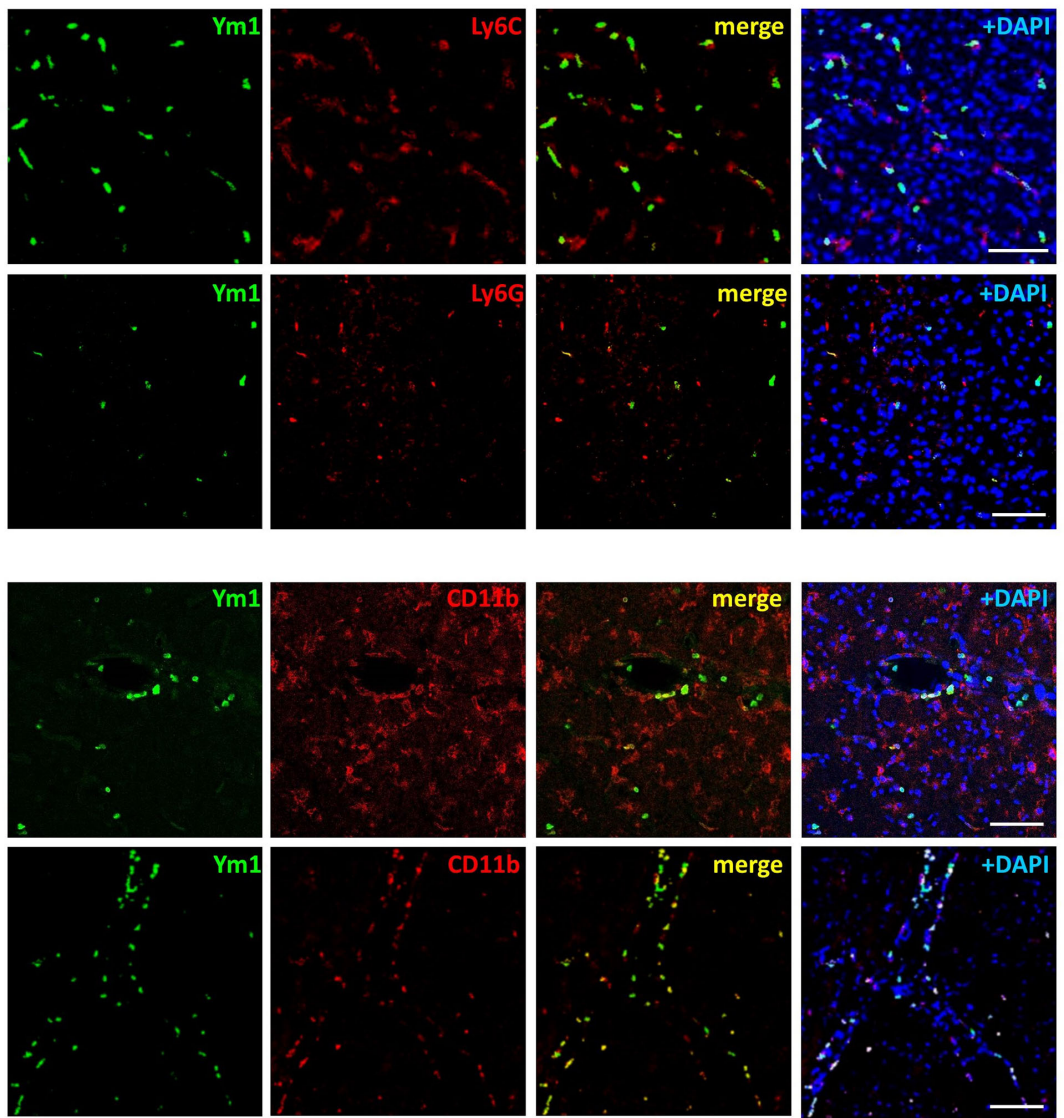
Ym1 is expressed in myeloid cells infiltrating from the periphery, resembling monocytes/macrophages and neutrophils. Representative immunofluorescence images showing Ym1 expression (green fluorescence) in CD11b immunopositive myeloid cells (lining the blood vessels and populating the perivascular space), in Ly-6C immunopositive monocytes/macrophages and in Ly-6G immunopositive neutrophils. Nuclei are counterstained with DAPI (blue signal). Scale bars = 50 μm.

In order to clarify the significance of this latter finding, we analysed the expression of Ym1 in the brain cortex of mice. As compared with sham tissue, exposure to 15-min or 1-h MCAo elevated Ym1 protein levels in the ipsilateral cortex (**Figure 3**). This effect was further potentiated in the animals undergone ischemic PC before the more severe ischemia (i.e., PC+MCAo group, **Figure 3**). By immunofluorescence analysis (**Figure 3**), we observed that the signal corresponding to CD11b immunopositive myeloid cells was significantly (P=0.0286, Mann Whitney test, n=4 mice per experimental group) increased in the cortex of animals pre-exposed to PC before MCAo (66.94 ± 1.56 CD11b^+^ cells/0.31 mm^2^) as compared with MCAo alone (44.90 ± 11.18 CD11b^+^ cells/0.31 mm^2^). Amoeboid-shaped myeloid cells express Ym1 (arrows in **Figure 3**), likely corresponding to microglia/macrophages, since Ym1 signal was also detected in amoeboid Iba1 immunopositive cells (arrows in **Figure 3**). Interestingly, Ym1 immuno-signal almost completely overlapped with Ly-6B.2 (**Figure 3**), a glycophosphatidylinositol-anchored, heavily glycosylated protein expressed on myeloid cells, including Ly-6G+ neutrophils, Ly-6C+ inflammatory monocytes and some activated macrophages (58–60). Accordingly, Ym1-expressing cells mainly represent Ly6C+ monocytes/macrophages and Ly-6G+ neutrophils (**Figure 4**). The latter evidence, together with the presence of Ym1 signal in amoeboid-shaped CD11b+ myeloid cells lining the endothelium and populating perivascular regions (**Figure 4**), strongly suggest their recruitment from the periphery.

**Figure 5:**
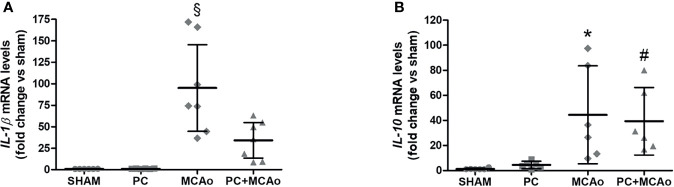
Ischemic PC prevents elevation of *IL-1β* mRNA levels, while not affecting elevation of *IL-10* mRNA levels caused by 1h MCAo in the ipsilateral cortex of mice. Quantitative PCR was used to quantify mRNA levels of **(A)** *IL-1β* and **(B)** *IL-10* in the ipsilateral cortex of mice subjected to SHAM surgery, ischemic PC (15 min MCAo followed by 72h of reperfusion), 1h MCAo followed by 24h of reperfusion (MCAo) or PC+MCAo. Data were analysed by one-way ANOVA followed by Tukey’s *post-hoc* test (^§^P = 0.0001 vs SHAM and P = 0.0001 vs PC and P = 0.0063 vs MCAo; *P = 0.0173 vs SHAM and P = 0.0290 vs PC; ^#^P = 0.0401 vs SHAM; n = 6-7 mice per experimental group).

These finding demonstrate that ischemic PC is associated with spleen contraction and elevation of Ym1 expression in this lymphoid organ, which correspond to increased brain recruitment of M2-like myeloid cells. This latter evidence was coincident with reduced inflammatory reactions in the ischemic hemisphere, since ischemic PC prevented elevation of mRNA levels of the pro-inflammatory cytokine IL-1β in the ipsilateral cortex (**Figure 5**). By contrast, brain elevation of the mRNA levels of IL-10 induced by MCAo, was unaffected by previous exposure to ischemic PC (**Figure 5**), which is consistent with the elevation of M2-like anti-inflammatory phenotypes (**Figure 3**).

**Figure 6:**
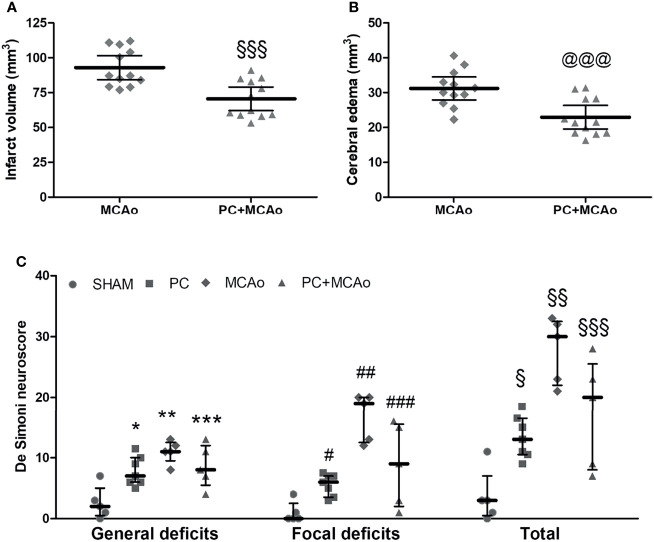
Ischemic PC significantly reduces brain damage and neurological deficits caused by 1h MCAo. **(A)** Cerebral infarct volume and **(B)** brain edema in mice subjected to 1h MCAo followed by 24h of reperfusion preceded 72h before by sham surgery (MCAo) or by ischemic PC (PC+MCAo); ^§§§^P = 0.0005 and ^@@@^P = 0.0009 vs MCAo (unpaired t-test, n = 12 mice per experimental group). **(C)** General, focal and total deficits caused in mice by SHAM surgery, ischemic PC (15min MCAo, followed by 72h of reperfusion), 1h MCAo followed by 24h of reperfusion (MCAo) or ischemic PC 72h before MCAo (PC+MCAo). Data were analysed by Kruskal-Wallis test followed by Bonferroni’s correction (*P = 0.0227 vs corresponding SHAM, **P = 0.0088 vs corresponding SHAM and P = 0.0182 vs corresponding PC, ***P = 0.0212 vs corresponding SHAM, ^#^P = 0.0111 vs corresponding SHAM, ^##^P = 0.0080 vs corresponding SHAM and P = 0.0044 vs corresponding PC, ^###^P = 0.0339 vs corresponding SHAM, ^§^P = 0.0145 vs corresponding SHAM, ^§§^P = 0.0088 vs corresponding SHAM and P = 0.0045 vs corresponding PC, ^§§§^P = 0.0278 vs corresponding SHAM; n = 5-7 mice per experimental group).

Overall, the increased anti-inflammatory/pro-inflammatory ratio observed in the PC+MCAo group corresponded to reduced brain infarct volume (**Figure 6**) and ischemic edema (**Figure 6**) as compared with 1h MCAo. Histological protection was associated with amelioration of functional outcome, since both focal and general deficits caused by 1h MCAo were prevented by ischemic PC (**Figure 6**).

## Discussion

The results of the present work confirm previous findings demonstrating that PC by short-term focal ischemia protects the brain and reduces neurological deficits when applied 72h before severe MCAo in rats (64–66) or mice (12, 25, 48, 49). Moreover, we extend previous observations by demonstrating that ischemic PC attenuates cellular (i.e., elevation of circulating inflammatory myeloid cells) and soluble (i.e., elevation of plasma miR-329-5p) inflammatory responses triggered by a severe ischemic insult. In the periphery, we originally observed elevation of the M2/N2 marker Ym1 in the spleen after PC (regardless of whether it was followed by 1h MCAo) that was coincident with spleen contraction. In fact, pre-exposure to ischemic PC potentiated the ischemia-induced elevation of Ym1 in the lesioned cortex, where Ym1 immunopositive signal was detected in amoeboid myeloid cells infiltrating from the periphery, likely representing monocytes/macrophages and neutrophils. This was coincident with reduction of pro/anti-inflammatory ratio in the brain, since pre-exposure to PC prevented elevation of *IL-1β* mRNA induced by 1h MCAo, while it did not affect ischemia-induced elevation of *IL-10* mRNA levels in the ipsilateral ischemic cortex.

The development of ischemic tolerance has been associated with a significant transcriptomic response to ischemic PC (12, 25, 48, 67). Our findings are in line with the evidence that the transcriptional response to severe ischemia is dominated by cerebral upregulation of genes that coordinate immune responses and host defence; whereas, when ischemia is preceded by ischemic PC this response is reprogrammed and returns to basal levels (12). Toll-like receptors (4 and 9) and downstream cerebral activation of inflammatory reactions, including microglia stimulation, Type-I interferon pathways and release of inflammatory cytokines such as TNF-α, have been reported to underlie protection by ischemic PC in rodents (26, 36, 68).

Using TargetScan database (www.targetscan.org), we performed a reverse target prediction analysis to identify miR-329-5p based on its potential to regulate inflammatory and immune mediators, including TLR-4, IL-1 and TNF-related signalling pathways, with a relevant role in ischemia and PC. Our findings demonstrate that ischemic PC prevents elevation of miR-329-5p occurring in the plasma of mice subjected to 1-h MCAo. This suggests that reduced inflammatory status in the periphery underlies neuroprotection by PC. In fact, miR-329 has been implicated in the modulation of inflammatory responses during ischemia, since knockdown of its expression inhibits the release of TNF-α and nitric oxide in the supernatant of OGD-stimulated microglial cells. The underlying mechanism involves the long noncoding RNA (lncRNA) small nucleolar RNA host gene 1 (SNHG1) with relevant neuroprotective and immunomodulatory functions (69–72). Moreover, miR-329 downregulates the expression of its target gene *TGF-β1* (73) that codes for a cytokine with known anti-inflammatory and beneficial functions in ischemic stroke (74, 75). MiR-329 also regulates TLR4/tumor necrosis factor receptor associated factor 6 (TRAF6)/nuclear factor (NF)-κB signaling (76), a pathway involved in ischemic damage and preconditioning, as well as in the regulation of neutrophil dynamics (26, 77–79). Our finding that miR-329 plasma levels modulation by PC/MCAo is paralleled by similar fluctuations in the number of circulating inflammatory myeloid cells, is in line with the evidence that proatherogenic cells in the circulation (i.e., inflammatory Ly-6C^hi^ monocytes, neutrophils, and blood platelets) were decreased upon miRNA-329 inhibition (80). Moreover, increased expression of miR-329 in peripheral blood mononuclear cells (PBMCs) of the patients with coronary artery disease may lead to progression of atherosclerosis plaque (81).

A recent *in vivo* microarray study has demonstrated that ischemic PC triggers in cortical microglia a transcriptomic profile dominated by upregulation of cellular proliferation genes that is coincident with the time of peak neuroprotection in the mouse brain (25). Accordingly, proliferation of myeloid cells underlies protection against ischemic injury (31), while elevation of infiltrating myeloid cells and fractalkine-dependent microglia proliferation were observed in the ischemic hemisphere of preconditioned mice (25). Fractalkine signalling attenuates inflammatory responses in microglia and promotes M1-to-M2 polarization in other experimental contexts (82, 83), suggesting that these pathways may also be shared by preconditioning. The latter hypothesis is supported by our evidence that ischemic PC enhances the expression of the M2 marker Ym1 in amoeboid-shaped myeloid cells populating the ischemic hemisphere, as also previously reported (84–86). Actually, the majority of Ym1-expressing cells were Ly-6B.2 immunopositive, thus corresponding to monocytes/macrophages and Ly-6G+ neutrophils, likely infiltrating from the periphery. Thus, our findings strongly suggest that an increased recruitment of M2/N2-polarized innate immune cells underlies neuroprotection exerted by ischemic PC in mice. This extends previous evidence documenting that monocytes isolated and adoptively transferred from lipopolysaccharide (LPS)-preconditioned mice confer protection against a prolonged subsequent MCAo (41). Cell-tracking studies have revealed that these protective monocytes are mobilized from the spleen and reach the brain and meninges, where they mitigate inflammation and neutrophil influx induced by ischemia (41).

In the blood of mice subjected to transient MCAo we observed elevation of Ly-6G+ and Ly-6C+ inflammatory leukocytes after 24h of reperfusion. Both effects were attenuated by ischemic PC and coincided with reduced brain damage and inflammation. This is consistent with the evidence that higher leukocytes and neutrophils blood counts are associated with larger infarct volumes (87) and with poor functional outcomes in acute ischemic stroke patients with neurological deterioration (88). Neutrophils are among the first peripheral immune cells recruited to the ischemic brain, entering the tissue through inflamed blood vessels, soft meninges or the choroid plexus. Previous work has documented that the peak elevation of neutrophil count in blood of mice subjected to 60-min MCAo occurs 24h after injury, whereas their infiltration to the brain can be observed from 12 h after stroke, reaching a maximum after 1-3 days (89, 90). In patients, higher neutrophil counts are associated with more severe strokes at admission (91) and larger infarct volumes (87); while elevated neutrophil-to-lymphocyte (NLR) ratio is significantly associated with poor prognosis (92) and risk of hemorrhagic transformation (93). Although blockade of their recruitment has been shown to be neuroprotective in models of acute brain injury (94), suggesting their potential to aggravate damage, neutrophils also display beneficial roles (77, 95, 96). This latter evidence does not seem to apply to PC, since we observed that elevation of the number of circulating neutrophils induced after MCAo is prevented by the neuroprotective ischemic PC. Similarly, LPS preconditioning reduced neutrophil composition in the blood after transient MCAo compared with non-preconditioned control mice, which was suggested to contribute to reduced infiltration in the ischemic brain (97)

Besides neutrophils, circulating monocytes are recruited by monocyte chemoattractant protein (MCP)-1 and extensively infiltrate into the ischemic parenchyma, reaching a peak 2-3 days after the insult (90, 98). A higher monocyte blood count has been associated with ischemic stroke severity and adverse prognosis of patients (99–102). This is coherent with the elevated number of Ly-6C+ monocytes that we observed in the blood of mice subjected to transient MCAo, an effect that is attenuated by pre-exposure to the neuroprotective PC stimulus. Accordingly, Ly-6C^high^ monocytes are increased in the blood of mice subjected to transient MCAo (103). Once recruited to the brain, Ly-6C^high^ monocytes can mature into macrophages bearing M2-like phenotypes (104–106). By contrast, Ly-6C^low^ patrolling monocytes are redundant in this experimental context, since they do not affect progression and/or recovery after ischemic stroke (107). In human, classical CD14+ monocytes secrete IL-1β, TNF-α and IL-6 to exert a pro-inflammatory effect (108) and their number increases in the blood of acute stroke patients, being independently associated with poor outcome (109, 110); whereas, nonclassical and intermediate CD16+ monocytes display an inverse correlation with mortality and poor functional and histological outcomes (110).

Bona fide undifferentiated monocytes are present in a reservoir within the red pulp of the spleen and, upon distal tissue injury (i.e., myocardial ischemia), they can be mobilized and migrate to the damaged site to promote damage or healing (41, 62, 63). Splenic atrophy is considered a hallmark of post-stroke peripheral immune activation and, in agreement with previous evidence (111–113), we detected reduced spleen size after 24h of reperfusion as compared with sham surgery. Splenic contraction occurs by 3 hours until 7 days after transient focal cerebral ischemia in mice and is accompanied by monocytes mobilization and migration to the stroked brain (114, 115). Splenic atrophy was believed to affect ischemic brain injury by exacerbating the inflammatory response though the release of spleen-derived immune cells into the circulation, their migration to the brain and the activation of microglial cells (111, 112, 116–120). Various data confirm the existence of a correlation between ischemia-induced splenic atrophy and the histological and functional outcome (54, 121, 122). Nevertheless, in some contexts, spleen size does not directly reflect mobilization of cells to the brain, but it is indeed correlated with the inflammatory cerebral milieu (123). In fact, post-stroke spleen contraction was accompanied by decreased number of Ly-6C^high^ and Ly-6C^low^ subsets in the spleen that temporally coincided with respective increases in the ischemic brain (114). Although this latter effect was prevented by splenectomy, infarct size and swelling were not reduced in the asplenic mice, strongly supporting a distinct effect of the two monocyte subtypes in stroke outcome (114). In fact, a negative correlation between splenic macrophage, T, and B cells and the neurological deficit score suggests that these splenic immune cells may aid in stroke recovery (62). Accordingly, the spleen was required to unleash the neuroprotective capacity of adoptively transferred LPS-preconditioned monocytes (41), thus suggesting that homing to the spleen may be necessary to acquire the reparative phenotype. Under our experimental conditions, exposure to PC (followed or not by MCAo) resulted in significant elevation of Ym1 expression in the mouse spleen, despite its reduced weight vs sham (strongly suggesting elevated M2/M1 or N2/N1 ratios), that was coincident with elevated brain levels of M2-polarized myeloid cells recruited to the ischemic hemisphere. The evidence that the number of circulating neutrophils and monocytes is reduced in the PC+MCAo experimental group may reflect the fact that although immune cells may migrate to the brain through blood circulation, they may also traffic through other means such as lymphatic vessels; thus the blood does not necessarily reflect migration of immune cells from the periphery to the brain. However, confirmation of our hypothesis would require demonstration of the effects of adoptively transferred M2/N2 myeloid cells in asplenic animals: the lack of such evidence represents a major limitation of our work.

Genes associated with alternative macrophage polarization, including Ym1 (*Chi3l1*), *IL-10* and *Arginase 1*, are up-regulated in LPS-preconditioned monocytes that exert protective roles in mice subjected to MCAo (41). In the meninges, LPS-primed monocytes suppress post-ischemic expression of inflammatory cytokines involved in leukocytes trafficking to the brain, thus resulting in decreased immune cell (mainly neutrophils) accumulation in the ischemic brain (41). In line with this observation, we observe that ischemic PC is associated with elevated brain expression of the M2 marker Ym1, predominantly expressed in myeloid cells likely infiltrating from the periphery, but also in amoeboid-shaped Iba1-immunopositive microglia/macrophages. As brain resident immune cells, microglia exert a dualistic role in ischemic stroke, displaying pleiotropic functions depending on their phenotypes: pro-inflammatory, or ‘M1’ phenotype prevailing in the acute stage, and beneficial ‘M2’ phenotypes occurring at later stages after the insult. The latter phagocytose non viable, necrotic tissue, and set the stage for reparative processes such as the restoration of synapses, angiogenesis, neurogenesis and gliogenesis (38, 124). Microarray data demonstrate that the transcriptomic response of preconditioned cortical microglia is dominated by upregulation of genes involved in cell cycle activation and cellular proliferation (36). In particular, ischemic PC triggers microglia proliferation in the ipsilateral cortex in the absence of tissue infarction, a process dependent on signalling through the fractalkine receptor CX3CR1 (25). This latter finding strongly suggests the occurrence of polarization towards M2-like reparative phenotypes, since aberrant activation of microglia toward a neurotoxic profile was observed in mice lacking CX3CR1 (29, 125). Moreover, fractalkine was shown to promote the M1-to-M2 shift of microglia phenotype (83). Accordingly, hypoxic preconditioning attenuates ischemia-induced inflammatory reactions and elevates the ratio of M2/M1 polarization markers of microglia in rat brain (126). Moreover, after ischemic PC, both microglia and peripheral immune cells (i.e., monocytes and neutrophils) are increased in the ischemic cortex (25, 37). CCR2-dependent brain infiltration of LPS-preconditioned monocytes that acquire an alternatively activated phenotype, has been reported to provide neuroprotection in mice undergone MCAo, likely trough their production of IL-10 and activation of i-NOS (41). Interestingly, CCR2 is the major receptor involved in brain infiltration of monocytes triggered by ischemia and plays a crucial role in their repairing functions (103, 104, 127, 128). The fact that hypoxic/ischemic PC is also dependent on CCL2/CCR2 signalling (129, 130), together with our evidence of increased brain infiltration of Ym1 immunopositive myeloid cells following PC, strongly suggest that cerebral recruitment of beneficial/reparative monocytes may represent a general mechanism of ischemic tolerance.

Thus, it is intriguing to speculate that modulation of the peripheral immune system towards protective phenotypes may represent a crucial endogenous mechanism triggered by ischemic preconditioning to protect against ischemic brain damage and inflammation. In fact, the elevation of *IL-1β* expression caused by transient MCAo in the ischemic cortex is prevented by ischemic PC, thus suggesting that tolerance is associated with attenuation of the inflammatory and detrimental effects of this cytokine in the lesioned tissue (131–135).

Moreover, our findings are consistent with the elevation of IL-10 protein levels observed in the rodent brain after 24h of permanent or transient MCAo (136, 137), with neurons being the major source of this cytokine and its receptor at this time-point (137). Since LPS-induced PC was associated with elevation of IL-10 levels in mouse blood and in preconditioned monocytes (41, 138), while hypoxic PC promotes the expression of IL-10 in the ischemic cortex of rats subjected to transient focal ischemia (126), we were expecting elevation of this cytokine following brain recruitment of M2 monocytes in the ischemic hemisphere. However, we found that *IL-10* mRNA levels did not change in the brain of PC+MCAo experimental group as compared to MCAo. This evidence, together with the effect on brain mRNA levels of *IL-1β* strongly suggest that ischemic PC exerts protection by reducing the ratio pro-inflammatory/anti-inflammatory responses. In fact, both *in vitro* and *in vivo* hypoxia/ischemia models have revealed that IL-10 exerts neuroprotective effects by mitigating the production of inflammatory cytokines and by inhibiting the activation and brain recruitment of detrimental immune cells (139). Our hypothesis is further supported by the evidence that either chemical and ischemic preconditioning was shown to attenuate ischemia-induced mRNA elevations of inflammatory cytokines (i.e., IL-1β and IL-6), while not affecting the elevation of the protective TGF-β occurring in the ischemic cortex (66).

In conclusion, our data demonstrate that ischemic PC attenuates the elevation of cellular (i.e., monocytes and neutrophils) and soluble (i.e., miR-329-5p) inflammatory mediators triggered by severe ischemia in mouse blood. Moreover, when 1h MCAo was preceded by the PC stimulus, spleen contraction was associated with increased levels of the M2/N2 marker Ym1 that coincided with elevated brain levels of Ym1-immunopositive innate immune cells, including amoeboid microglia/macrophages, monocytes and neutrophils. The elevation of these anti-inflammatory phenotypes produced by PC in the ischemic hemisphere was coincident with reduced pro-inflammatory/anti-inflammatory ratio (measured as *IL-1β*/*IL-10* mRNA levels) and in amelioration of histological and functional outcomes after 24h of reperfusion. These data reaffirm the crucial role of the peripheral innate immune system in the progression of cerebral ischemic damage and highlight the possibility of targeting these endogenous peripheral responses to develop novel effective stroke therapies.

The fact that the present study was conducted in young adult, male mice limits its relevance to an exploratory phase. In fact, the mechanisms implicated in tolerance induced by ischemic PC require validation in larger, more clinically relevant contexts, including both sexes, aged animals, comorbidity (i.e., hypertension and diabetes), at least two species and results from multiple centres, as recently recommended by the Stroke Treatment Academic Industry Roundtable (140).

## Data Availability Statement

The original contributions presented in the study are included in the article. Further inquiries can be directed to the corresponding author.

## Ethics Statement

The animal study was reviewed and approved by OPBA of the Department of Pharmacy, Health and Nutritional Sciences, *via* Savinio, Ed. Polifunzionale, I-87036 Rende (CS), Italy, and by the Committee set by the Ministry of Health at the National Institute of Health (Rome) (Prot. n. 975/2017-PR and 701/2020-PR).

## Author Contributions

DA designed the study, wrote the manuscript and contributed to acquisition, analysis and interpretation of data. DR, MF, FG, CS, MP, and GP contributed to data acquisition and analysis. GB supervised the work and edited the manuscript. All authors contributed to the article and approved the submitted version.

## Funding

This work was supported by the Italian Ministry of Education, University and Research (PRIN codes 2015KRYSJN to DA and 2017XKJTLW_001 to GB; L. 232/2016: Excellence awarded to the Department of Pharmacy, Health and Nutritional Sciences).

## Conflict of Interest

The authors declare that the research was conducted in the absence of any commercial or financial relationships that could be construed as a potential conflict of interest.

## Publisher’s Note

All claims expressed in this article are solely those of the authors and do not necessarily represent those of their affiliated organizations, or those of the publisher, the editors and the reviewers. Any product that may be evaluated in this article, or claim that may be made by its manufacturer, is not guaranteed or endorsed by the publisher.
